# Ferrofluid lubrication of circular squeeze film bearings controlled by variable magnetic field with rotations of the discs, porosity and slip velocity

**DOI:** 10.1098/rsos.170254

**Published:** 2017-12-13

**Authors:** Rajesh C. Shah, Rajiv B. Shah

**Affiliations:** Department of Applied Mathematics, Faculty of Technology and Engineering, The Maharaja Sayajirao University of Baroda, Vadodara 390 001, Gujarat, India

**Keywords:** ferrofluid, Shliomis model, squeeze film bearings, lubrication, porous disc, variable magnetic field

## Abstract

Based on the Shliomis ferrofluid flow model (SFFM) and continuity equation for the film as well as porous region, modified Reynolds equation for lubrication of circular squeeze film bearings is derived by considering the effects of oblique radially variable magnetic field (VMF), slip velocity at the film–porous interface and rotations of both the discs. The squeeze film bearings are made up of circular porous upper disc of different shapes (exponential, secant, mirror image of secant and parallel) and circular impermeable flat lower disc. The validity of Darcy's Law is assumed in the porous region. The SFFM is important because it includes the effects of rotations of the carrier liquid as well as magnetic particles. The VMF is used because of its advantage of generating maximum field at the required active contact area of the bearing design system. Also, the effect of porosity is included because of its advantageous property of self-lubrication. Using Reynolds equation, general form of pressure equation is derived and expression for dimensionless load-carrying capacity is obtained. Using this expression, results for different bearing design systems (due to different shapes of the upper disc) are computed and compared for variation of different parameters.

## 1. Introduction

A ferrofluid (FF) or magnetic fluid (MF) is a colloidal dispersion of magnetic particles in a non-conducting carrier liquid. Neuringer–Rosensweig (NR) [1] suggested FF flow model in which only magnetic body force is considered without any effects of rotations of the carrier liquid as well as magnetic particles. With the invention of FF [1], its applications as lubricant on various bearing design systems have been found from different viewpoints [2,3]. Everywhere it was shown that the better performances of the bearing characteristics were obtained. The NR model depends on the assumption that the magnetization vector is parallel to the magnetic field vector. Prajapati [2] studied effect of MF on different porous squeeze film bearing designs like circular, annular, elliptic, conical, etc. It was concluded that the load-carrying capacity increases with the increase of magnetization parameter. Montazeri [3] numerically discussed FF lubricated hydrodynamic journal bearings. It was shown that compared to conventional lubricant, FF improves hydrodynamic characteristics and provides a higher load capacity with the reduction in friction coefficient.

In the case of different angular velocities of rotations of the carrier liquid as well as magnetic particles, frictional forces arise. These forces cause an increase in the effective viscosity of the FF and it has major impact on the pressure when FF is used as lubricant. Shliomis [4] considered rotations of the carrier liquid as well as magnetic particles in the FF flow model with magnetic body force. Many authors [5–14] studied this model from different viewpoints. Shukla & Kumar [5] analysed FF lubricated slider and squeeze film bearings using uniform transverse magnetic field by neglecting relaxation time of particle rotation. In their study they derived pressure equation under the assumptions that the FF is saturated (so that the saturation magnetization is independent of the applied magnetic field) and the magnetic moment relaxation time is negligible. However, Shah & Bhat [6] derived pressure equation without above assumptions of [5] in their study on FF squeeze film between curved annular plates. It was concluded that the load-carrying capacity and approaching time of squeeze films can be enhanced by increasing the volume concentration of solid phase in FF and the intensity of external magnetic fields. Shah [7] extended the above analysis [6] with the insertion of rotation effect of the upper plate, and studied different shapes (secant, exponential and flat) of the upper plate. The results showed that load-carrying capacity and response time increase with the increase of volume fraction of the particles and rotation of the upper plate. Also, it was shown that load-carrying capacity and response time increase with the increase of curvature of the exponential plate, whereas they decrease with the increase of curvature of the secant shape. Singh & Gupta [8] studied FF lubricated curved slider bearing with the effect of transverse magnetic field, and showed the improvement in stiffness and damping capacities due to the effects of rotation and volume concentration of magnetic particles. Lin [9] derived Reynolds equation for MF lubricated slider bearings using transverse magnetic field and showed the improvement in load-carrying capacity, dynamic stiffness and damping characteristics. Patel & Deheri [10] discussed FF lubrication of squeeze film in rotating rough curved circular discs with assorted porous structures. It was concluded that even if suitable magnetization is in force, roughness aspect must be accorded priority while designing the bearing system. Shah & Parikh [11] analysed FF lubrication of different shapes of slider bearings and compared dimensionless load-carrying capacity for the effect of squeeze velocity. It was concluded that the load-carrying capacity of all bearings remains constant with the increase of Langevin's parameter, whereas it has an increasing tendency with the increase of volume concentration of the particles. Lin *et al.* [12] studied effects of circumferential and radial rough surfaces on a non-Newtonian MF lubricated squeeze film. It was concluded that circumferential roughness effect increases the mean load-carrying capacity and lengthens the mean approaching time when compared with smooth discs. However, the radial roughness pattern showed the reverse trend. Huang & Wang [13] presented comprehensive review on FFs lubrication with some experimental studies. Nargund & Asha [14] studied load-carrying capacity of hyperbolic slider bearings and showed the better performance of the system.

All above studies based on the Shliomis model are with transverse magnetic field. It is observed that the study with oblique radially variable magnetic field (VMF) considering the effects of porosity, slip velocity at the film–porous interface and rotations of both the discs, is ignored. The Shliomis model is important because it includes the effects of rotations of the carrier liquid as well as magnetic particles, and it behaves differently in the case of VMF. The VMF is important because of having its advantage of generating maximum field at the required active contact area in the bearing design system. Also, looking to industrial applications, the above three effects are also important. The effect of porosity is included because of its advantageous property of self-lubrication. Hence, there is a need of the present paper. Thus, the aim of the present paper is to study lubrication of circular squeeze film bearings using the Shliomis FF flow model with the effects of oblique radially VMF, porosity, slip velocity at the film–porous interface and rotations of both the discs. The squeeze film bearings are made up of circular porous upper disc of different shapes (exponential, secant, mirror image of secant and parallel) and circular impermeable flat lower disc. While deriving the modified Reynolds equation, the validity of Darcy's Law is assumed in the porous region (matrix or layer). The continuity equation is also used in the film as well as porous region. Using this Reynolds equation, general form of pressure equation is derived and expression for dimensionless load-carrying capacity is obtained. Using this expression, results for different bearing design systems (due to different shapes of the upper disc) are computed and compared for variation of different parameters. The pressure equation derived in the present case is more general in nature and different from all previous studies. Moreover, the present analysis considers the effect of sample magnetic field and it can be extended to other forms of fields similarly. Also, the mirror image of secant squeeze film bearing is introduced for the first time in this paper. The symbols used in the paper are defined in table 1.

**Table 1.** Nomenclature.

*a*	radius of the circular discs (m)
FF	ferrofluid
*h*_0_	central film thickness (m)
*h*	film thickness defined in equations (2.1)–(2.4) (m)
${\dot{h}}_{0}$	squeeze velocity, $\text{d}h_{0}\text{/d}t$ (m s^−1^)
*H*	magnetic field strength (A m^−1^)
***H***	magnetic field vector
H*	thickness of the porous matrix (m)
*I*	sum of moments of inertia of the particles per unit volume (N s^2^ m*^−^*^2^)
*K*	quantity defined in equation (2.12) (A m*^−^*^4^)
*k*_B_	Boltzmann constant ($\text{J}\;{\text{°K}}^{-1}$)
*k*	permeability of the porous matrix (m^2^)
*m*	magnetic moment of a particle (A m^2^)
***M***	magnetization vector
*M*_0_	equilibrium magnetization (A m^−1^)
MF	magnetic fluid
*n*	number of particles per unit volume (m^−3^)
*p*	film pressure (N m^−2^)
*P*	fluid pressure in the porous matrix (N m^−2^)
***q***	fluid velocity vector
*r*	radial coordinate (m)
*s*	slip constant (m^−1^)
*t*	time (s)
*T*	temperature (°K)
*V*_sq_	dimensionless squeeze velocity parameter defined in equation (3.1)
VMF	variable magnetic field
*W*	load-carrying capacity (N)
$\bar{W}$	dimensionless load-carrying capacity defined in equation (3.9)
${\bar{W}}_{e}$	dimensionless load-carrying capacity for exponential squeeze film bearing
${\bar{W}}_{s}$	dimensionless load-carrying capacity for secant squeeze film bearing
${\bar{W}}_{is}$	dimensionless load-carrying capacity for mirror image of secant squeeze film bearing
${\bar{W}}_{p}$	dimensionless load-carrying capacity for parallel squeeze film bearing
*z*	axial coordinate (m)
Greek symbols	
*θ*	inclination of the magnetic field vector to the radial direction
*α*	curvature of the mirror image of secant upper disc (m^−2^)
*β*	curvature of the exponential upper disc (m^−2^)
*γ*	curvature of the secant upper disc (m^−2^)
$\bar{\alpha }$	*α a*^2^, dimensionless curvature parameter defined in equation (4.1*c*)
$\bar{\beta }$	*β a*^2^, dimensionless curvature parameter defined in equation (4.1*a*)
$\bar{\gamma }$	*γ a*^2^, dimensionless curvature parameter defined in equation (4.1*b*)
η	viscosity of the suspension (N s m^−2^)
$\eta_{0}$	viscosity of the carrier liquid (N s m^−2^)
$\eta_{r}$	porosity of the porous matrix in *r*-direction
$\mu_{0}$	free space permeability (N A^−2^)
*ξ*	dimensionless field strength (Langevin's parameter)
*ρ*	fluid density (N s^2^ m^−4^)
$\tau_{B}$	Brownian relaxation time (s)
$\tau_{s}$	magnetic moment relaxation time (s)
*φ*	volume concentration of the particles
*ψ*	dimensionless porous thickness parameter defined in equation (3.1)
$\Omega_{f}$	dimensionless rotational parameter defined in equation (3.1)
$\Omega_{u}$	rotational velocity of the upper disc (rad s^−1^)
$\Omega_{l}$	rotational velocity of the lower disc (rad s^−1^)
$\Omega_{r}$	$\Omega_{u}-\Omega_{l}$

## 2. Analysis

**Figure 1.**
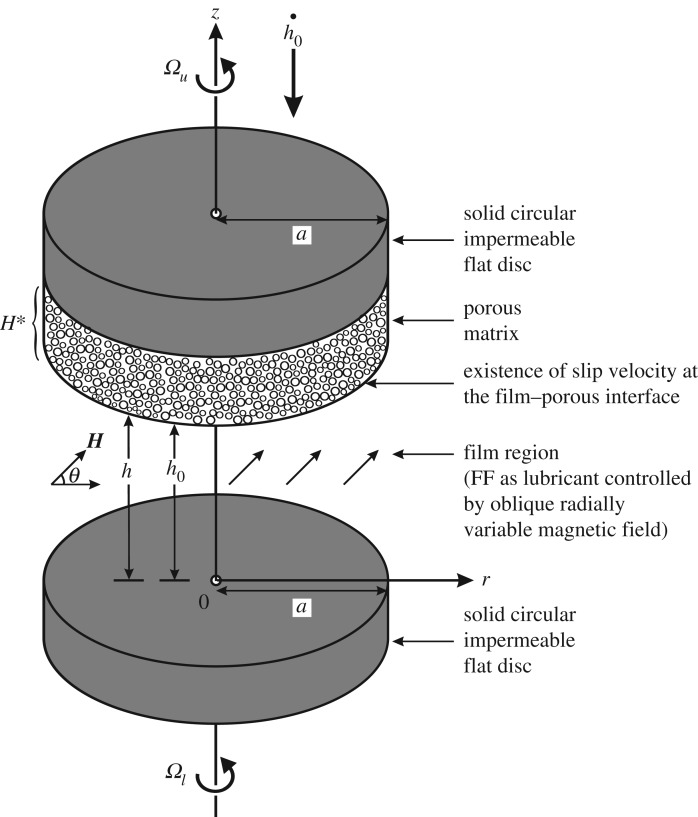
Schematic diagram of the physical configuration of the circular squeeze film bearing (case of both flat discs).

**Figure 2.**
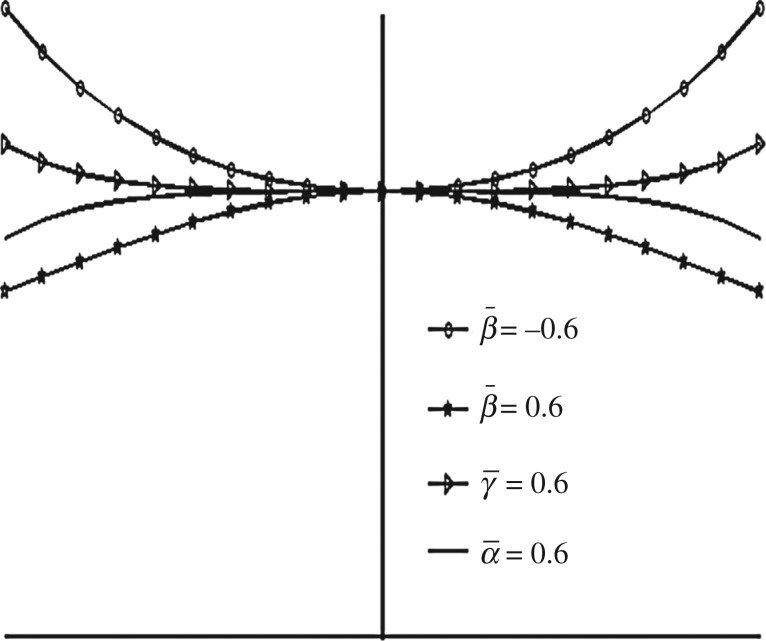
Exponential (${\bar{h}}_{e}={\text{e}}^{-\bar{\beta }R^{2}}$), secant (${\bar{h}}_{s}=\sec\;(\bar{\gamma }R^{2})$) and mirror image of secant (${\bar{h}}_{is}=2-\sec\;(\bar{\alpha }R^{2})$) shapes of the upper disc for $\bar{\beta }=-0.6,\bar{\beta }=\bar{\gamma }=\bar{\alpha }=0\text{.6}$.

Figure 1 shows the physical configuration of the problem under consideration, which consists of two circular discs (case of both flat discs is shown) each of radius *a*. The upper disc is made by attaching a porous matrix of uniform thickness H* to the solid impermeable disc. The upper disc may also be curved (which may be either exponential or secant or mirror image of secant shapes as shown in figure 2) while the lower disc is solid impermeable flat. The central film thickness is *h*_0_. The region between two discs is known as film region (lubrication region), which is filled with FF. The upper disc moves normally towards lower one with a uniform velocity, known as squeeze velocity ${\dot{h}}_{0}=\text{d}h_{0}\text{/d}t$, where *t* is time. The upper and lower discs rotated with rotational (angular) velocities *Ω_u_* and *Ω_l_*, respectively. Owing to the different shapes of the upper disc, the film thickness *h* takes following different forms.

Case 1. For exponentially curved upper disc

2.1 $$h=h_{0}{\text{e}}^{-\beta r^{2}};0\leq r\leq a,$$

where *β* is curvature and *r* is the radial coordinate.

Case 2. For secant curved upper disc

2.2 $$h=h_{0}\sec\;(\gamma r^{2});0\leq r\leq a,$$

where *γ* is curvature.

Case 3. For mirror image of secant curved upper disc

2.3 $$h=2h_{0}-h_{0}\sec\;(\alpha r^{2});0\leq r\leq a,$$

where *α* is curvature.

Case 4. For parallel upper disc

2.4 $$h=h_{0};0\leq r\leq a.$$

With reference to the shape of the upper disc, the bearing designs for the Cases 1–4 are referred to here as exponential squeeze film bearing, secant squeeze film bearing, mirror image of secant squeeze film bearing and parallel squeeze film bearing, respectively.

Neglecting inertia terms, assuming steady flow and other usual assumptions of lubrication, the basic flow equations governed by the Shliomis model [4,6,7] using cylindrical frame of reference can be written as follows.

*Equation of motion*

2.5 $$-\nabla p+\eta \nabla^{2}q+\mu_{0}(M∙\nabla )H+\frac{1}{2}\mu_{0}\nabla \times (M\times H)=\left(-\frac{\rho {\text{v}}^{2}}{r},0,0\right),$$

*Equation of magnetization*

2.6 $$M=\frac{M_{0}}{H}(H+\bar{\tau }\bar{\Omega }\times H)\text{,}\;\bar{\tau }=\frac{\tau_{B}}{1+(\mu_{0}M_{0}H\tau_{B}\tau_{s}/I)},$$

*Equation of continuity*

2.7 ∇∙q=0,

*Maxwell equations*

2.8 ∇×H=0

and

2.9 ∇∙(M+H)=0,

where *p* is the film pressure, *η* is the viscosity of the suspension, ***q*** is the fluid velocity vector, *µ*_0_ is the permeability of free space, ***M*** is the magnetization vector, ***H*** is the applied magnetic field vector, *ρ* is the fluid density, *v* is the tangential component of ***q***, *M*_0_ is the equilibrium magnetization, *H* is the magnitude of ***H***, $\bar{\Omega }=\frac{1}{2}\nabla \times q$, *τ*_B_ is the Brownian relaxation time, *τ_s_* is the magnetic moment relaxation time and *I* is the sum of moments of inertia of the particles per unit volume.

Also,

2.10 $$q=(\dot{r},r\dot{\theta },\dot{z})=(u,r\text{v},w),$$

where (r,θ,z) are cylindrical polar coordinates and dot (⋅) represents derivative with respect to *t*.

Assuming the predomination of the velocity gradient across the film, *v* as a linear function of the axial coordinate *z*, and the axially symmetric flow in the film as well as magnetic field, the *r*-component of equation (2.5) with the help of equation (2.6) and $\bar{\Omega }=\frac{1}{2}\nabla \times q$ implies

2.11 $$\frac{\partial^{2}u}{\partial z^{2}}=\frac{1}{\eta (1+(\mu_{0}M_{0}H\bar{\tau }/4\eta ))}\left[\frac{\text{d}p}{\text{d}r}-\mu_{0}M_{0}\frac{\text{d}H}{\text{d}r}-\rho r{\left(\frac{z}{h}\Omega_{r}+\Omega_{l}\right)}^{2}\right],$$

where $\Omega_{r}=\Omega_{u}-\Omega_{l}$ and *u* is the radial component of ***q***. The inclination *θ* of H=H(r)(cos⁡θ,0,sin⁡θ),θ=θ(r,z) to the radial direction is assumed to be small and can be obtained from condition (2.8).

In order to consider active contact area in the neighbourhood of *r* = 2*a*/3, the magnetic field strength of radially VMF should be chosen (referring to [15]) as

2.12 $$H=Kr^{2}(a-r)\text{,}$$

where *K* is the quantity chosen to suit the dimensions of both sides of equation (2.12). Such a field attains maximum at *r* = 2*a*/3 and vanishes at *r* = 0 and *r* = *a*. For other active contact areas, suitable form of magnetic field strength should be chosen.

Defining the following quantities for a suspension of spherical particles [4,6]

2.13 $$M_{0}=\text{nm}\left(\text{coth}\;\xi -\frac{1}{\xi }\right),H=\frac{k_{B}T\xi }{\mu_{0}m},\tau_{B}=\frac{3\eta V}{k_{B}T},V=\frac{\varphi }{n},\tau_{s}=\frac{I}{6\eta \varphi },\tau =\frac{3}{2}\varphi \frac{\xi -\text{tanh}\;\xi }{\xi +\text{tanh}\;\xi },$$

equation (2.11) takes the form

2.14 $$\frac{\partial^{2}u}{\partial z^{2}}=\frac{1}{\eta (1+\tau )}\left[\frac{\text{d}}{\text{d}r}\left(p-nk_{B}T\ln\frac{\sinh\xi }{\xi }\right)-\rho r{\left(\frac{z}{h}\Omega_{r}+\Omega_{l}\right)}^{2}\right],$$

where *n* is the number of magnetic particles per unit volume, *m* is the magnetic moment of a particle, *ξ* (Langevin's parameter) is the dimensionless form of *H*, *k*_B_ is the Boltzmann constant, *T* is the temperature and *φ* is the volume concentration of the particles.

Solving equation (2.14) using slip boundary conditions [15,16]

2.15 $$u=0\;\text{when}\;z=0,\;u=-\frac{1}{s}\frac{\partial u}{\partial z};s=\frac{5}{\sqrt{k\eta_{r}}},\;\text{when}\;z=h$$

yields

2.16 $$u=\frac{1}{2\eta (1+sh)(1+\tau )}\left[\begin{array}{l} \{(1+sh)z^{2}-h(2+sh)z\}\frac{\text{d}}{\text{d}r}\left(p-nk_{B}T\ln\frac{\sinh\xi }{\xi }\right)\\ -\frac{\rho r}{6h^{2}}\left\{\begin{array}{l} \{(1+sh)z^{4}-h^{3}(4+sh)z\}\Omega_{r}^{2}+4h\{(1+sh)z^{3}-h^{2}(3+sh)z\}\Omega_{r}\Omega_{l}\\ \;+\;6h^{2}\{(1+sh)z^{2}-h(2+sh)z\}\Omega_{l}^{2} \end{array}\right\} \end{array}\right],$$

where *k* is permeability of the porous matrix, $\eta_{r}$ is porosity of the porous matrix in *r*-direction and *s* is the slip constant.

Substituting equation (2.16) into the integral form of continuity equation (2.7) in cylindrical polar coordinates for the film region

2.17 $$\frac{1}{r}\frac{\partial }{\partial r}\int_{0}^{h}(ru)dz+w_{h}-w_{0}=0;\;w_{h}=w{|}_{z=h},w_{0}=w{|}_{z=0},$$

yields

2.18 $$\frac{1}{r}\frac{\text{d}}{\text{d}r}\left[\frac{-h^{3}}{12\eta (1+sh)\;(1+\tau )}\left\{\begin{array}{l} (4+sh)r\frac{\text{d}}{\text{d}r}\left(p-nk_{B}T\ln\frac{\sinh\xi }{\xi }\right)\\ \;-\;\frac{\rho r^{2}}{10}\{(18+3sh)\Omega_{u}^{2}+(14+4sh)\Omega_{u}\Omega_{l}+(8+3sh)\Omega_{l}^{2}\} \end{array}\right\}\right]+w_{h}=0,$$

where *w* is the axial velocity component of ***q*** and *w*_0_ = 0 as the lower disc is impermeable.

Moreover, the relation between viscosity of the suspension *η* and viscosity of the carrier liquid *η*_0_ is given by [4,6]

2.19 $$\eta =\eta_{0}\left(1+\frac{5}{2}\varphi \right).$$

Assuming the validity of Darcy's Law, the radial and axial components (considering the contributions from the magnetic pressure and rotation of the upper disc) of the fluid velocity in the porous matrix yield, respectively, as

2.20 $$\bar{u}=-\frac{k}{\eta }\left[\frac{\partial }{\partial r}\left(P-nk_{B}T\ln\frac{\sinh\xi }{\xi }\right)-\rho r\Omega_{u}^{2}-\frac{1}{4}\frac{\partial }{\partial z}\left(\mu_{0}M_{0}H\bar{\tau }\frac{\partial u}{\partial z}\right)\right]$$

and

2.21 $$\bar{w}=-\frac{k}{\eta }\left[\frac{\partial }{\partial z}\left(P-nk_{B}T\ln\frac{\sinh\xi }{\xi }\right)+\frac{1}{4r}\frac{\partial }{\partial r}\left(r\mu_{0}M_{0}H\bar{\tau }\frac{\partial u}{\partial z}\right)\right],$$

where *P* is the fluid pressure in the porous matrix.

Substituting equations (2.20) and (2.21) in the continuity equation for the porous matrix

2.22 $$\frac{1}{r}\frac{\partial }{\partial r}(r\bar{u})+\frac{\partial \bar{w}}{\partial z}=0$$

and integrating it across the porous matrix (*h*, *h* + H*) yields

2.23 $${\frac{\partial }{\partial z}\left(P-nk_{B}T\ln\frac{\sinh\xi }{\xi }\right)|}_{z=h}=\frac{H^{*}}{r}\frac{\text{d}}{\text{d}r}\left\{r\frac{\text{d}}{\text{d}r}\left(p-nk_{B}T\ln\frac{\sinh\xi }{\xi }\right)\right\}-2H^{*}\rho \Omega_{u}^{2},$$

where Morgan–Cameron approximation [15] and the fact that the surface *z* = *h* + H* is impermeable is used.

Owing to continuity of the fluid velocity components across the film–porous interface,

2.24 $$w_{h}={\dot{h}}_{0}+{\bar{w}}_{h}.$$

Using equations (2.16), (2.21), (2.23), (2.24), equation (2.18) yields the Reynolds equation for the present study as

2.25 $$\begin{array}{rl} & \frac{1}{r}\frac{\text{d}}{\text{d}r}\left[\left\{12kH^{*}+\frac{h^{3}(4+sh)+6ks\tau h^{2}}{\left(1+sh)\;(1+\tau \right)}\right\}r\frac{\text{d}}{\text{d}r}\left(p-nk_{B}T\ln\frac{\sinh\xi }{\xi }\right)\right]\\ & \;=\frac{1}{r}\frac{\text{d}}{\text{d}r}\left[\begin{array}{l} (6\eta {\dot{h}}_{0}+12\rho kH^{*}\Omega_{u}^{2})r^{2}+\frac{\rho (3\Omega_{u}^{2}+2\Omega_{u}\Omega_{l}+\Omega_{l}^{2})ks\tau r^{2}h^{2}}{\left(1+sh)(1+\tau \right)}\\ \;+\;\frac{\rho r^{2}h^{3}\{(18+3sh)\Omega_{u}^{2}+(14+4sh)\Omega_{u}\Omega_{l}+(8+3sh)\Omega_{l}^{2}\}}{10(1+sh)(1+\tau )} \end{array}\right]. \end{array}$$

## 3. Solution

Introducing dimensionless quantities

3.1 $$\begin{array}{rl} \bar{p} & =-\frac{h_{0}^{3}p}{\eta a^{2}{\dot{h}}_{0}},\;R=\frac{r}{a},\;\psi =\frac{kH^{*}}{h_{0}^{3}},\;\bar{h}=\frac{h}{h_{0}},\;\bar{s}=sh_{0},\;\delta =\frac{6k}{h_{0}^{2}},\;\\ V_{\mathrm{sq}} & =-\frac{{\dot{h}}_{0}}{\Omega_{u}h_{0}},\;S=\frac{\rho \Omega_{u}h_{0}^{2}}{\eta V_{\mathrm{sq}}},\;\Omega_{f}=\frac{\Omega_{l}}{\Omega_{u}},\;\mu^{*}=-\frac{nk_{B}Th_{0}^{3}}{\eta a^{2}{\dot{h}}_{0}} \end{array}$$

and using equation (2.12), equation (2.25) becomes

3.2 $$\frac{1}{R}\frac{\text{d}}{\text{d}R}\left[GR\frac{\text{d}}{\text{d}R}\left(\bar{p}-\mu^{*}\ln\frac{\sinh\xi }{\xi }\right)\right]=\frac{1}{R}\frac{\text{d}}{\text{d}R}(RF),$$

where

3.3 $$\begin{array}{rl} G & =12\psi +\frac{{\bar{h}}^{3}(4+\bar{s}\bar{h})+\delta \bar{s}\tau {\bar{h}}^{2}}{\left(1+\bar{s}\bar{h})\;(1+\tau \right)}, \end{array}$$

3.4 $$\begin{array}{rl} F & =(-6+12\psi S)R+\frac{\delta \bar{s}\tau {\bar{h}}^{2}SR(3+2\Omega_{f}+\Omega_{f}^{2})}{6(1+\bar{s}\bar{h})\;(1+\tau )}\\ & \;+\frac{SR{\bar{h}}^{3}\{(18+3\bar{s}\bar{h})+(14+4\bar{s}\bar{h})\Omega_{f}+(8+3\bar{s}\bar{h})\Omega_{f}^{2}\}}{10(1+\bar{s}\bar{h})\;(1+\tau )}, \end{array}$$

3.5 $$\begin{array}{rl} \xi & =\lambda R^{2}(1-R), \end{array}$$

3.6 $$\begin{array}{rl} \text{and}\;\lambda & =\frac{\mu_{0}mKa^{3}}{k_{B}T}. \end{array}$$

Solving equation (3.2) using boundary conditions

3.7 $$\bar{p}\text{( 1) }=0,\frac{\text{d}\bar{p}}{\text{d}R}=0\;\text{when}\;R=0,$$

yields

3.8 $$\bar{p}=\mu^{*}\ln\frac{\sinh\xi }{\xi }+\int_{1}^{R}\frac{F}{G}\text{d}R.$$

The load-carrying capacity *W* of the bearing can be expressed in dimensionless form as

3.9 $$\bar{W}=-\frac{h_{0}^{3}W}{2\pi \eta a^{4}{\dot{h}}_{0}}=\int_{0}^{1}R\bar{p}dR=\mu^{*}I^{*}-\frac{1}{2}\int_{0}^{1}\frac{R^{2}F}{G}\text{d}R,$$

where

3.10 $$I^{*}=\frac{\lambda }{2}\int_{0}^{1}R^{3}(2-3R)\left(\frac{1}{\xi }-\coth\xi \right)\text{d}R,$$

and *G* and *F* are given by equations (3.3) and (3.4), respectively.

## 4. Results and discussion

The results for the dimensionless load-carrying capacity $\bar{W}$ given by equation (3.9) are computed using Simpson's one-third rule with step size 0.1. The representative values of the different parameters taken in computations are as follows [6,16,17]. These values remain fixed unless and until the calculation of $\bar{W}$ is made with respect to the variation of the particular parameter.

$$\begin{array}{rl} & a=0.05\;\text{m},\;h_{0}=0.00005\;\text{m},\;k_{B}=1.38\times 10^{-23}\text{J}{(}^{o}{\text{K) }}^{-1},\\ & T=297{\;}^{o}\text{K},\;\mu_{0}m=1.75\times 10^{-25}\;\text{J}\;{\text{A}}^{-\text{1}}\text{m, }\\ & \varphi =0.0075,\;V=1.02\times 10^{-25}{\text{m}}^{3},\;\eta_{0}=0.012\;\text{N}\;\text{s}\;{\text{m}}^{-2},\\ & {\dot{h}}_{0}=-0.001\;\text{m}\;{\text{s}}^{-1},\;H\approx O(10^{3}),\;\rho =1400\;\text{N}\;{\text{s}}^{2}\;{\text{m}}^{-4},\;\eta_{r}=0.25\\ \text{and}\; & k=5.1\times 10^{-11}{\text{m}}^{2},\;H^{*}=0.000007\;\text{m} \end{array}$$

with the relations

$$\lambda =\frac{27}{4}\xi_{\max},K=1.26\times 10^{9}\xi_{\max}.$$

Also, for smaller values of *ξ*,

$$\begin{array}{rl} & \coth\xi -\frac{1}{\xi }\to 0\\ \text{and}\; & \frac{\xi -\tanh\xi }{\xi +\tanh\xi }\to 0. \end{array}$$

The calculation of order of magnetic field strength is shown below.

From equation (2.12),

$$\begin{array}{rl} H_{\max} & =0.1852\times 10^{-4}K\\ \text{for }H & \approx O(10^{3}),\;K=10^{7}/0.1822. \end{array}$$

Using subscripts *e*, *s*, *is*, *p* for the concerned quantities when the squeeze film bearing designs are of exponential, secant, mirror image of secant and parallel shapes, respectively, equations (2.1)–(2.4) for computation become

4.1a $$\begin{array}{rl} \bar{h} & ={\bar{h}}_{e}={\text{e}}^{-\bar{\beta }R^{2}};\;\bar{\beta }=\beta a^{2},\;0\leq R\leq 1, \end{array}$$

4.1b $$\begin{array}{rl} \bar{h} & ={\bar{h}}_{s}=\sec\;(\bar{\gamma }R^{2});\;\bar{\gamma }=\gamma a^{2},\;0\leq R\leq 1, \end{array}$$

4.1c $$\begin{array}{rl} \bar{h} & ={\bar{h}}_{is}=2-\sec\;(\bar{\alpha }R^{2});\;\bar{\alpha }=\alpha a^{2},\;0\leq R\leq 1 \end{array}$$

4.1d $$\begin{array}{rl} \text{and}\;\bar{h} & ={\bar{h}}_{p}=1;\;0\leq R\leq 1. \end{array}$$

The sketch of the above different shapes (except for parallel; for parallel shape refer to figure 1) are shown in figure 2 for

$$\bar{\beta }=-0.6,\bar{\beta }=\bar{\gamma }=\bar{\alpha }=0\text{.6.}$$

The computed values of $\bar{W}$ for different parameters are displayed graphically. Before discussing graphs, it should be noted here that counterclockwise (positive) or clockwise rotations of either of the discs can be decided by looking from the top of the bearing system or with respect to the vertical axis.

**Figure 3.**
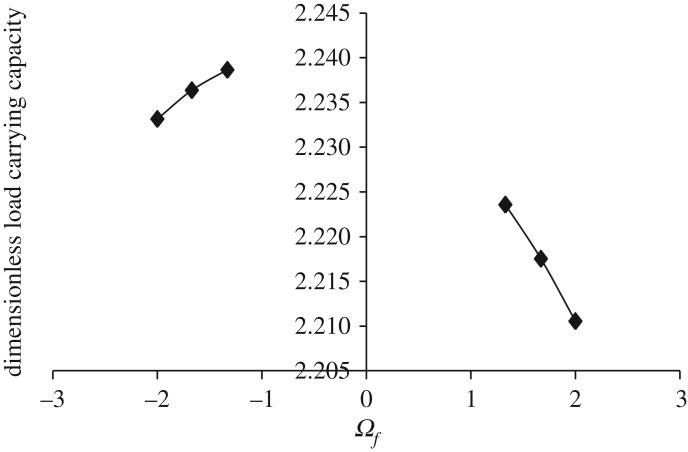
Variation in $\bar{W}$ for different values of $\Omega_{f}$ considering $|\Omega_{l}\;|\;>\;\Omega_{u}$ for $\bar{h}={\bar{h}}_{e}={\text{e}}^{-\bar{\beta }R^{2}}$ when $\Omega_{u}=30\pi$ and $\bar{\beta }=0.6$.

**Figure 4.**
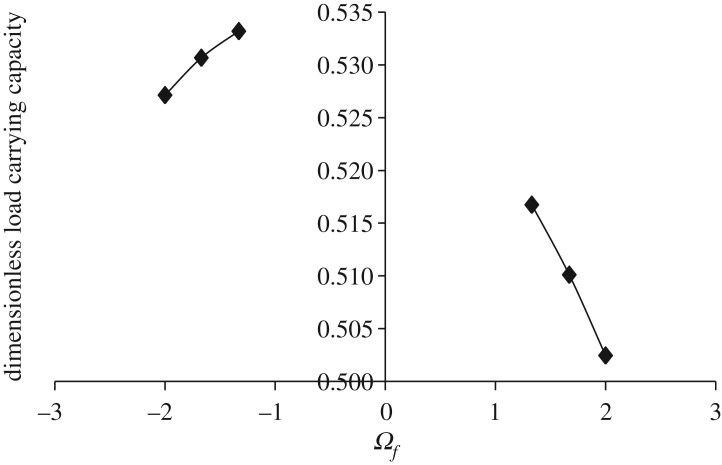
Variation in $\bar{W}$ for different values of $\Omega_{f}$ considering $|\Omega_{l}\;|\;>\;\Omega_{u}$ for $\bar{h}={\bar{h}}_{s}=\sec\;(\bar{\gamma }R^{2})$ when $\Omega_{u}=30\pi$ and $\bar{\gamma }=0.6$.

**Figure 5.**
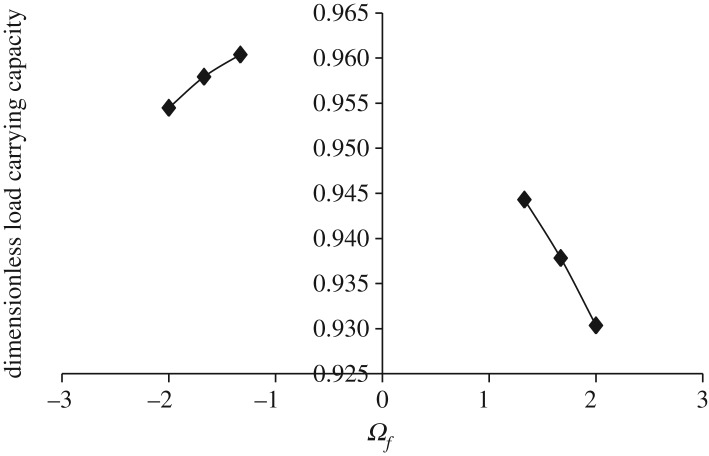
Variation in $\bar{W}$ for different values of $\Omega_{f}$ considering $|\Omega_{l}\;|\;>\;\Omega_{u}$ for $\bar{h}={\bar{h}}_{is}=2-\sec\;(\bar{\alpha }R^{2})$ when $\Omega_{u}=30\pi$ and $\bar{\alpha }=0.6$.

**Figure 6.**
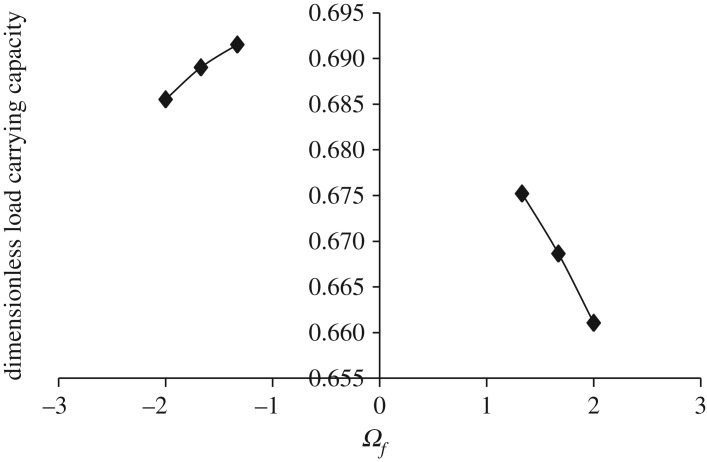
Variation in $\bar{W}$ for different values of $\Omega_{f}$ considering $|\Omega_{l}\;|\;>\;\Omega_{u}$ for $\bar{h}={\bar{h}}_{p}=1$ when $\Omega_{u}=30\pi$.

Figures 3–6 show the variation in $\bar{W}$ as a function of dimensionless rotational parameter $\Omega_{f}$ considering $|\Omega_{l}\;|\;>\;\Omega_{u}$ (that is, when the lower disc is rotated faster than the upper disc either in counterclockwise or clockwise direction) for different geometry of the squeeze film bearings like exponential, secant, mirror image of secant and parallel, respectively. The additional fixed values taken in computations are as follows:

(1) $\Omega_{u}=30\pi$, which indicates the rotation of the upper disc in counterclockwise direction with a fixed value 30π.
(2) Dimensionless curvature parameters $\bar{\beta }=\bar{\gamma }=\bar{\alpha }=0.6$.

It is observed, in general from figures 3–6, that $\bar{W}$ decreases in the case when $\Omega_{f}$ increases along the positive axis or decreases along the negative axis. That is, $\bar{W}$ decreases in the case when the speed of rotation of the lower disc increases more than 30π either in counterclockwise or clockwise direction. It is also observed that $\bar{W}$ is more in the case of clockwise rotation of the lower disc and maximum nearer to $\Omega_{f}=-1$. Moreover, this behaviour of $\bar{W}$ is same for all bearing designs. Table 2 shows the maximum value of $\bar{W}$, when the lower disc is rotated in different directions with speed 40π. It is observed that when the lower disc is rotated in clockwise direction, the increase rate of $\bar{W}$ is more and approximately 0.90% for exponential squeeze film bearing, 1.92% for secant squeeze film bearing, 2.13% for mirror image of secant squeeze film bearing and 1.47% for parallel squeeze film bearing. It should be noted here that the mirror image of secant squeeze film bearing design shape is introduced for the first time in the study because such type of shape exists in industry while manufacturing the disc.

**Table 2.** Values of $\bar{W}$ when lower disc is rotated in different directions either with $\Omega_{l}=-40\pi$ (clockwise) or $\Omega_{l}=40\pi$ (counterclockwise) considering $\Omega_{u}=30\pi$ and $\bar{\beta }=\bar{\gamma }=\bar{\alpha }=0.6$.

	$\Omega_{f}$	${\bar{W}}_{e}$	${\bar{W}}_{s}$	${\bar{W}}_{is}$	${\bar{W}}_{p}$
clockwise	−1.33	2.24	0.53	0.96	0.69
counterclockwise	1.33	2.22	0.52	0.94	0.68
% increase in $\bar{W}$		0.90	1.92	2.13	1.47

**Figure 7.**
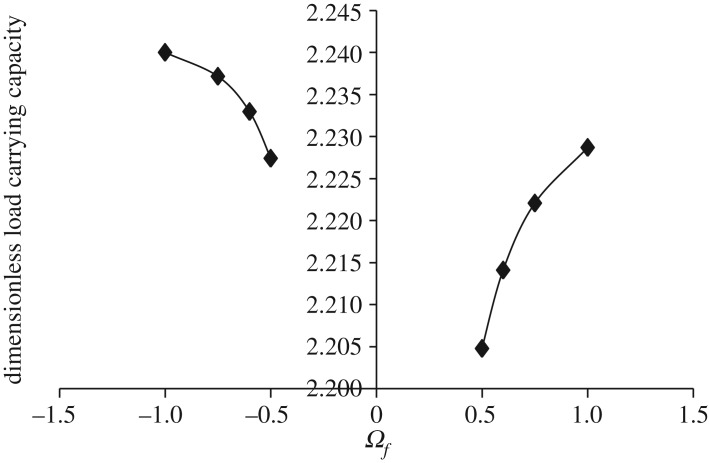
Variation in $\bar{W}$ for different values of $\Omega_{f}$ considering $\Omega_{l}\;\leq \;|\Omega_{u}|$ for $\bar{h}={\bar{h}}_{e}={\text{e}}^{-\bar{\beta }R^{2}}$ when $\Omega_{l}=30\pi$ and $\bar{\beta }=0.6$.

**Figure 8.**
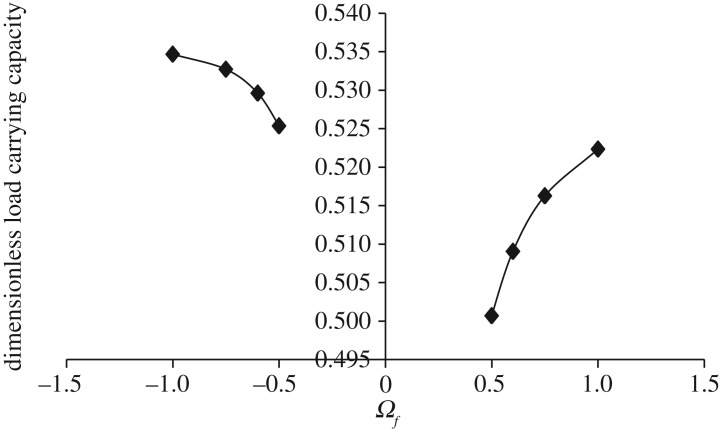
Variation in $\bar{W}$ for different values of $\Omega_{f}$ considering $\Omega_{l}\;\leq \;|\Omega_{u}|$ for $\bar{h}={\bar{h}}_{s}=\sec\;(\bar{\gamma }R^{2})$ when $\Omega_{l}=30\pi$ and $\bar{\gamma }=0.6$.

**Figure 9.**
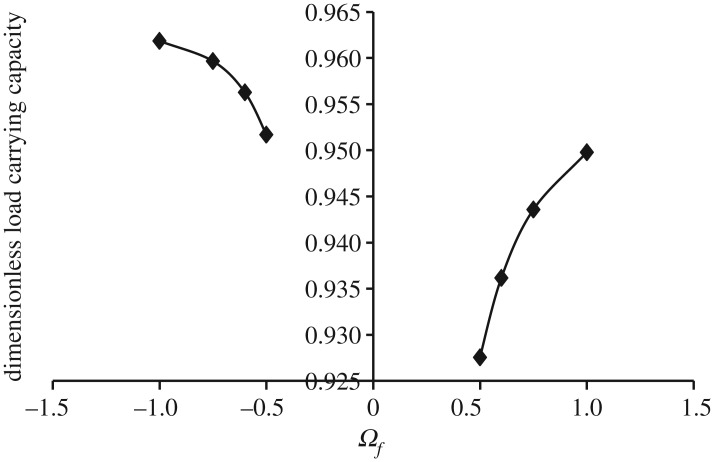
Variation in $\bar{W}$ for different values of $\Omega_{f}$ considering $\Omega_{l}\;\leq \;|\Omega_{u}|$ for $\bar{h}={\bar{h}}_{is}=2-\sec\;(\bar{\alpha }R^{2})$ when $\Omega_{l}=30\pi$ and $\bar{\alpha }=0.6$.

**Figure 10.**
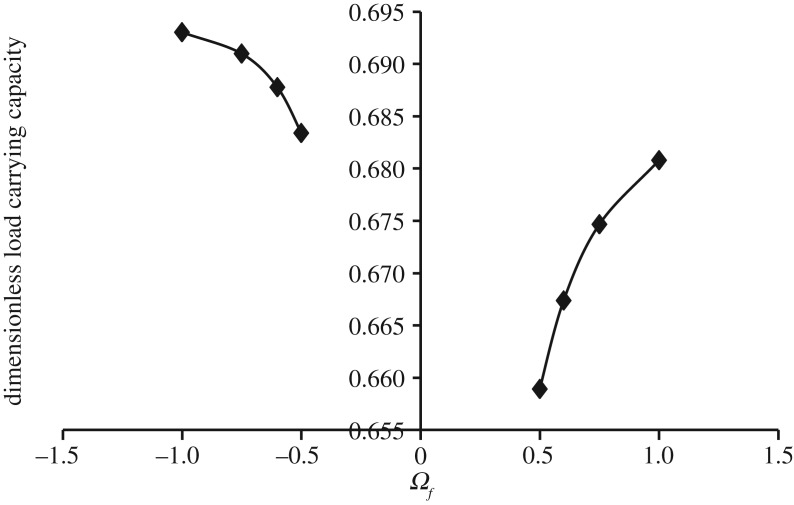
Variation in $\bar{W}$ for different values of $\Omega_{f}$ considering $\Omega_{l}\;\leq \;|\Omega_{u}|$ for $\bar{h}={\bar{h}}_{p}=1$ when $\Omega_{l}=30\pi$.

Figures 7–10 show the variation in $\bar{W}$ as a function of $\Omega_{f}$ considering $\Omega_{l}\;\leq \;|\Omega_{u}|$ (that is, when the upper disc is rotated faster or equal speed than the lower disc either in counterclockwise or clockwise direction) for different geometry of the squeeze film bearings like exponential, secant, mirror image of secant and parallel, respectively. Here, the additional fixed values taken as $\Omega_{l}=30\pi$ and $\bar{\beta }=\bar{\gamma }=\bar{\alpha }=0.6$. It is observed, in general, that $\bar{W}$ increases in the case when $\Omega_{f}$ increases along the positive axis or decreases along the negative axis. That is, $\bar{W}$ increases in the case when the speed of rotation of the upper disc moves from higher values to 30π either in counterclockwise or clockwise direction. It is also observed that $\bar{W}$ is more in the case of clockwise rotation of the upper disc and maximum at $\Omega_{f}=-1$. Moreover, this behaviour of $\bar{W}$ is same for all bearing designs. Table 3 shows the maximum value of $\bar{W}$, when the upper disc is rotated in different directions with speed 30π. It is observed that when the upper disc is rotated in clockwise direction, the increase rate of $\bar{W}$ is more and approximately 0.45% for exponential squeeze film bearing, 1.92% for secant squeeze film bearing, 1.05% for mirror image of secant squeeze film bearing and 1.47% for parallel squeeze film bearing.

**Table 3.** Values of $\bar{W}$ when upper disc is rotated in different directions either with $\Omega_{u}=-30\pi$(clockwise) or $\Omega_{u}=30\pi$(counterclockwise) considering $\Omega_{l}=30\pi$ and $\bar{\beta }=\bar{\gamma }=\bar{\alpha }=0.6$.

	$\Omega_{f}$	${\bar{W}}_{e}$	${\bar{W}}_{s}$	${\bar{W}}_{is}$	${\bar{W}}_{p}$
clockwise	−1.0	2.24	0.53	0.96	0.69
counterclockwise	1.0	2.23	0.52	0.95	0.68
% increase in $\bar{W}$		0.45	1.92	1.05	1.47

It should be noted here that for the same data values, counterclockwise rotation of the upper disc and clockwise rotation of the lower disc, or clockwise rotation of the upper disc and counterclockwise rotation of the lower disc, gives the same results due to kinematics of the rotation. That is, when $\Omega_{u}=-30\pi$ and $\bar{\beta }=\bar{\gamma }=\bar{\alpha }=0.6$ are fixed, and when the lower disc is rotated faster than the upper disc either in clockwise or counterclockwise direction, then the same results are obtained as shown in figures 3–6 since $\Omega_{f}=\Omega_{l}/\Omega_{u}$. The same is also true for figures 7–10.

Since $\Omega_{f}=\Omega_{l}/\Omega_{u}$, when $\Omega_{f}=0$ (that is, when there is no rotation of the lower disc irrespective of the rotation of the upper disc in different directions), it is observed from table 4 that for all bearing designs, $\bar{W}$ decreases as speed of rotations of the upper disc increases. Again, in this calculation $\bar{\beta }=\bar{\gamma }=\bar{\alpha }=0.6$ is fixed.

**Table 4.** Effects on $\bar{W}$ when the rotation of the lower disc is zero (that is, $\Omega_{l}=0$) irrespective of the rotation of the upper disc in different directions (that is, either counterclockwise or clockwise) considering $\bar{\beta }=\bar{\gamma }=\bar{\alpha }=0.6$.

$\Omega_{u}$	30*π* (or −30*π*)	40*π* (or −40*π*)	50*π* (or −50*π*)	60*π* (or −60*π*)
${\bar{W}}_{e}$	2.2385	2.2338	2.2277	2.2203
${\bar{W}}_{s}$	0.5331	0.5291	0.5239	0.5176
${\bar{W}}_{is}$	0.9603	0.9561	0.9507	0.9441
${\bar{W}}_{p}$	0.6914	0.6874	0.6821	0.6757

Table 5 represents the results of $\bar{W}$ when $\Omega_{f}$ takes negative values (that is, either $\Omega_{u}$ is rotated in counterclockwise direction and $\Omega_{l}$ is rotated in clockwise direction or $\Omega_{u}$ is rotated in clockwise direction and $\Omega_{l}$ is rotated in counterclockwise direction). Table 6 represents the results of $\bar{W}$ when $\Omega_{f}$ takes positive values (that is, either both the discs are rotated in counterclockwise direction or clockwise direction). For both the tables $\bar{\beta }=\bar{\gamma }=\bar{\alpha }=0.6$ is fixed. It is observed from both the tables that $\bar{W}$ decreases as the speed of rotations of both the discs increases. Further, $\bar{W}$ is more in the case when the speed of rotations of both the discs is in different directions to each other.

**Table 5.** Effects on $\bar{W}$ when $\Omega_{f}$ takes negative values (that is, either $\Omega_{u}$ is rotated in counterclockwise direction and $\Omega_{l}$ is rotated in clockwise direction or $\Omega_{u}$ is rotated in clockwise direction and $\Omega_{l}$ is rotated in counterclockwise direction) considering $\bar{\beta }=\bar{\gamma }=\bar{\alpha }=0.6$.

$\Omega_{u}$	30*π* (or −30*π*)	40*π* (or −40*π*)	50*π* (or −50*π*)	60*π* (or −60*π*)
$\Omega_{l}$	−30*π* (or 30*π*)	−40*π* (or 40*π*)	−50*π* (or 50*π*)	−60*π* (or 60*π*)
${\bar{W}}_{e}$	2.2400	2.2364	2.2318	2.2262
${\bar{W}}_{s}$	0.5347	0.5319	0.5283	0.5240
${\bar{W}}_{is}$	0.9619	0.9589	0.9550	0.9504
${\bar{W}}_{p}$	0.6930	0.6902	0.6865	0.6820

**Table 6.** Effects on $\bar{W}$ when $\Omega_{f}$ takes positive values (that is, either both the discs rotated in counterclockwise direction or in clockwise direction) considering $\bar{\beta }=\bar{\gamma }=\bar{\alpha }=0.6$.

$\Omega_{u}$	30*π* (or −30*π*)	40*π* (or −40*π*)	50*π* (or −50*π*)	60*π* (or −60*π*)
$\Omega_{l}$	30*π* (or −30*π*)	40*π* (or −40*π*)	50*π* (or −50*π*)	60*π* (or −60*π*)
${\bar{W}}_{e}$	2.2287	2.2163	2.2004	2.1810
${\bar{W}}_{s}$	0.5223	0.5100	0.4941	0.4746
${\bar{W}}_{is}$	0.9498	0.9374	0.9215	0.9021
${\bar{W}}_{p}$	0.6808	0.6684	0.6525	0.6331

While discussing figures 3–10 and tables 2–6, the following behaviours of $\bar{W}$ are observed in general.

(1) Maximum $\bar{W}$ is obtained in the case of exponential squeeze film bearing while minimum $\bar{W}$ in the case of secant squeeze film bearing. Thus, for all bearing designs $\bar{W}$ can be obtained in the order ${\bar{W}}_{e}>{\bar{W}}_{is}>{\bar{W}}_{p}>{\bar{W}}_{s}.$ This may be because of the following reason.

Referring to figure 2, in the case of exponential squeeze film bearing the curvature of the upper disc at the centre is in downward direction (that is, upper disc is concave with respect to lower disc), whereas in the case of secant shape the curvature is in upward direction (that is, upper disc is convex with respect to lower disc). Table 7 shows the behaviour of curvatures of all the upper discs by referring to figure 2. As the maximum magnetic field is taken at *r* = 2*a*/3, so because of such magnetic field and exponential shape of the upper disc, nearly closed bearing design system appears which leads to less possibility of leakage. Thus, maximum pressure generation is possible, which implies increase in $\bar{W}$. The curvature of the upper disc of mirror image of secant squeeze film bearing is less in downward direction when compared with exponential shape, so $\bar{W}$ is less in this case. Thus, as curvature of the upper disc at the centre increases in downward direction (that is, as concavity of the upper disc increases with respect to lower disc), $\bar{W}$ increases significantly. This may be the reason of obtaining $\bar{W}$ in the order ${\bar{W}}_{e}>{\bar{W}}_{is}>{\bar{W}}_{p}>{\bar{W}}_{s}.$

**Table 7.** Comparative chart of curvatures of the upper discs.

shape of the lower disc	shape of the upper disc	curvature of the upper disc	shape of the upper disc with respect to lower disc	curvature at the centre for concave discs	curvature at the centre for convex discs
parallel (flat)	exponential (refer equation (4.1*a*))	$\bar{\beta }>0$	concave	maximum	—
		$\bar{\beta }<0$	convex	—	maximum curvature in upward direction
	secant (refer equation (4.1*b*))	$\bar{\gamma }>0$ or $\bar{\gamma }<0$	always convex	—	less curvature in upward direction when compared with exponential disc
	mirror image of secant (refer equation (4.1*c*))	$\bar{\alpha }>0$ or $\bar{\alpha }<0$	always concave	less when compared with exponential shape	—
	parallel (refer equation (4.1*d*))	$\bar{\beta }=\bar{\gamma }=\bar{\alpha }=0$	parallel	—	—

(2) Maximum $\bar{W}$ is obtained in the case when both the discs are rotated in different directions to each other. This may be because of the following reason.

When the discs are rotated, then there is an appearance of centrifugal force (which points outward) in the rotating fluid system and it increases linearly with the radial distance *r*. In the case of rotation of both the discs in different directions to each other, the effect of centrifugal force is reduced (when compared with rotations of both the discs in the same direction) and as a result the leakage possibility is reduced. This may be because of twisted nature of the generated spikes. Nearly similar type of behaviour is obtained when only one disc is rotated.

**Figure 11.**
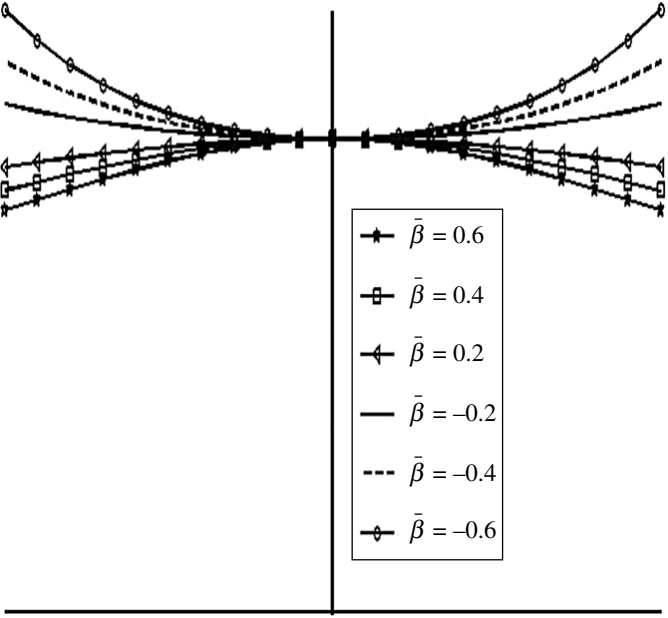
Different shapes of exponential upper disc for various values of dimensionless curvature parameter $\bar{\beta }$; $-0.6\leq \bar{\beta }\leq 0.6$.

**Figure 12.**
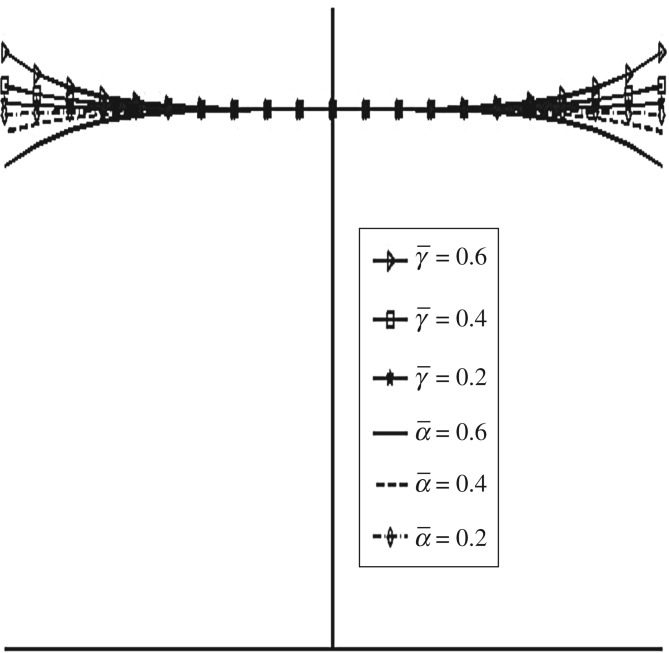
Different shapes of secant and mirror image of secant upper discs for various values of dimensionless curvature parameters $\bar{\gamma }(0.2\leq \bar{\gamma }\leq 0.6)$ and $\bar{\alpha }(0.2\leq \bar{\alpha }\leq 0.6)$, respectively.

Figure 11 shows different shapes of the exponential squeeze film bearing for $-0.6\leq \bar{\beta }\leq 0.6$. Here, the shape of the upper disc changes from convex to concave with respect to lower disc. Figure 12 shows various shapes of secant and mirror image of secant squeeze film bearings for $0.2\leq \bar{\gamma }\leq 0.6$ and $0.2\leq \bar{\alpha }\leq 0.6$, respectively. The upper disc of the secant squeeze film bearing takes convex shape, which shows less curvature to more curvature in upward direction as $\bar{\gamma }$ moves from 0.2 to 0.6. For the upper disc of mirror image of secant squeeze film bearing, concave shape is obtained which shows less curvature to more curvature in downward direction as $\bar{\alpha }$ moves from 0.2 to 0.6.

**Figure 13.**
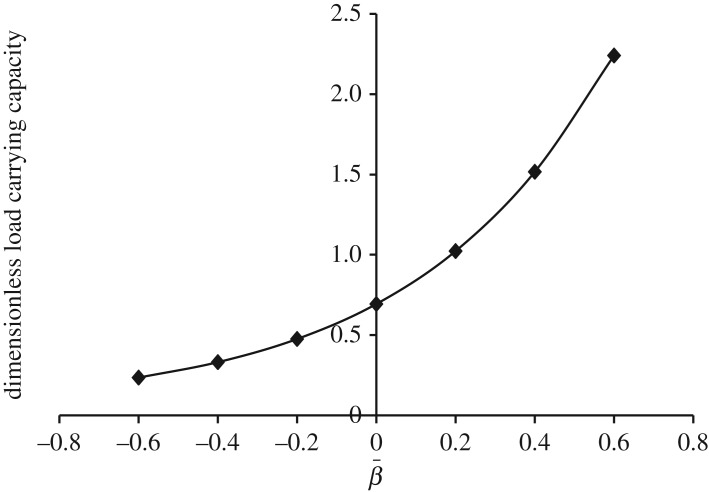
Variation in $\bar{W}$ for different values of dimensionless curvature parameter $\bar{\beta }$ and $\Omega_{f}=-1$ (that is, either $\Omega_{u}=30\pi$ and $\Omega_{l}=-30\pi$ or $\Omega_{u}=-30\pi$ and $\Omega_{l}=30\pi$) for $\bar{h}={\bar{h}}_{e}={\text{e}}^{-\bar{\beta }R^{2}}$.

**Figure 14.**
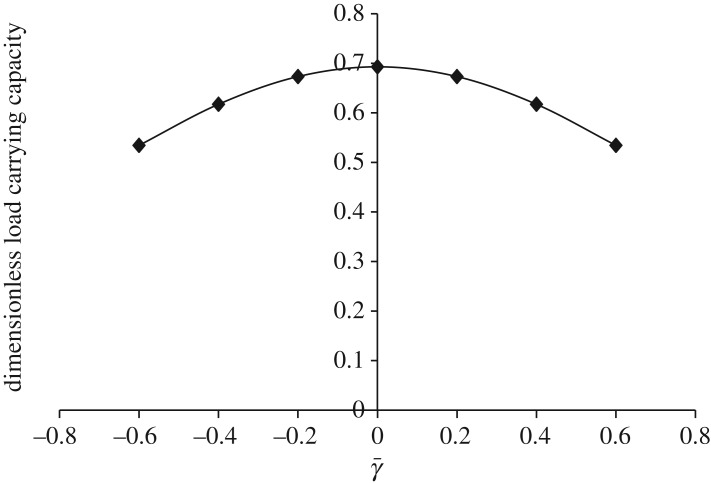
Variation in $\bar{W}$ for different values of dimensionless curvature parameter $\bar{\gamma }$ and $\Omega_{f}=-1$ (that is, either $\Omega_{u}=30\pi$ and $\Omega_{l}=-30\pi$ or $\Omega_{u}=-30\pi$ and $\Omega_{l}=30\pi$) for $\bar{h}={\bar{h}}_{s}=\sec\;(\bar{\gamma }R^{2})$.

**Figure 15.**
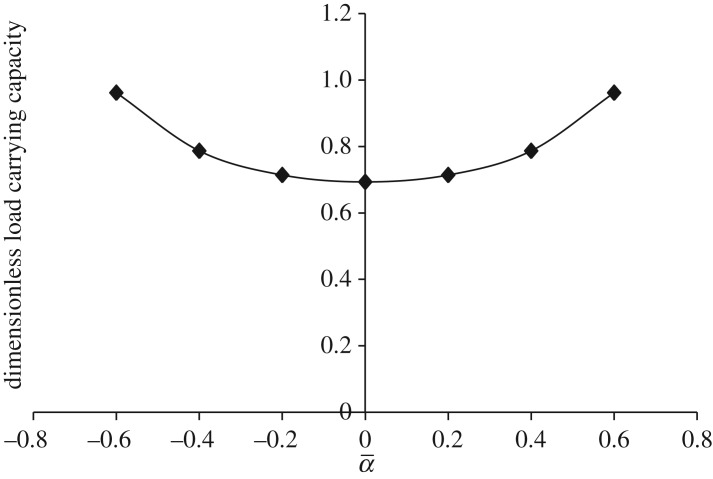
Variation in $\bar{W}$ for different values of dimensionless curvature parameter $\bar{\alpha }$ and $\Omega_{f}=-1$ (that is, either $\Omega_{u}=30\pi$ and $\Omega_{l}=-30\pi$ or $\Omega_{u}=-30\pi$ and $\Omega_{l}=30\pi$) for $\bar{h}={\bar{h}}_{is}=2-\sec\;(\bar{\alpha }R^{2})$.

**Figure 16.**
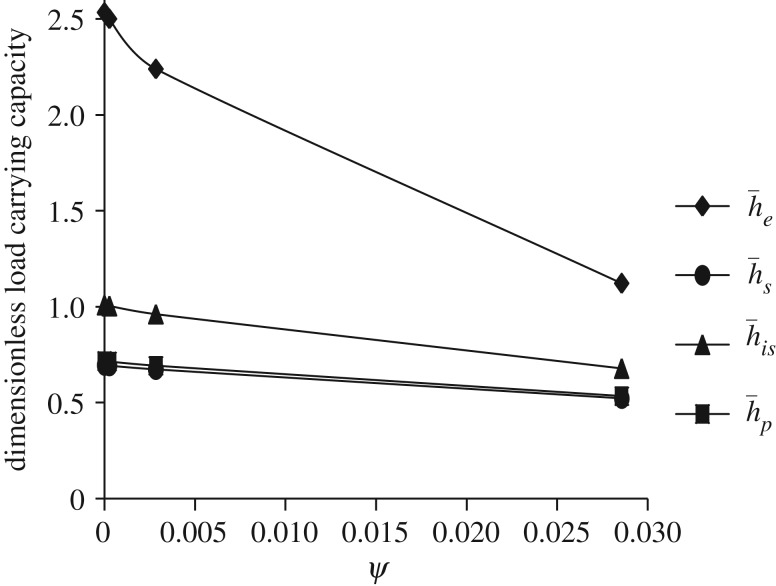
Variation in $\bar{W}$ for different values of dimensionless porous thickness parameter *ψ* and $\Omega_{f}=-1$ (that is, either $\Omega_{u}=30\pi$ and $\Omega_{l}=-30\pi$ or $\Omega_{u}=-30\pi$ and $\Omega_{l}=30\pi$) for all designs of $\bar{h}$ considering $\bar{\beta }=\bar{\alpha }=0.6$, $\bar{\gamma }=0.2$.

**Figure 17.**
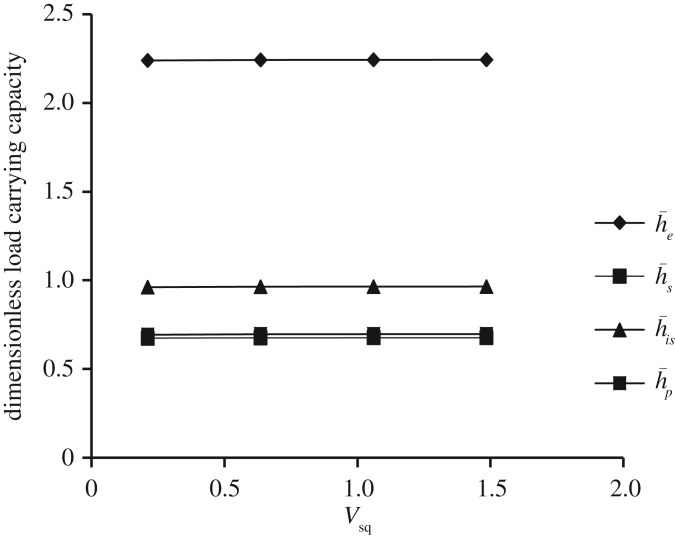
Variation in $\bar{W}$ for different values of dimensionless squeeze velocity parameter *V*_sq_ and $\Omega_{f}=-1$ (that is, either $\Omega_{u}=30\pi$ and $\Omega_{l}=-30\pi$ or $\Omega_{u}=-30\pi$ and $\Omega_{l}=30\pi$) for all designs of $\bar{h}$ considering $\bar{\beta }=\bar{\alpha }=0.6$, $\bar{\gamma }=0.2$.

Figures 13–17 show the variation in $\bar{W}$ when $\Omega_{f}=-1$ (that is, either $\Omega_{u}$ is rotated in counterclockwise direction and $\Omega_{l}$ is rotated in clockwise direction or $\Omega_{u}$ is rotated in clockwise direction and $\Omega_{l}$ is rotated in counterclockwise direction with a fixed value of 30π).

Figure 13 shows the variation in $\bar{W}$ as a function of dimensionless curvature parameter $\bar{\beta }$ for exponential squeeze film bearing. It is observed that $\bar{W}$ increases as $\bar{\beta }$ moves from − 0.6 to 0.6. That means concave shape with more curvature at the centre has more impact on the increase of $\bar{W}$. Figure 14 shows the variation in $\bar{W}$ as a function of dimensionless curvature parameter $\bar{\gamma }$ for secant squeeze film bearing. It is observed that $\bar{W}$ decreases as $\bar{\gamma }$ moves from 0.2 to 0.6. That means convex shape with less curvature at the centre in upward direction has more impact on the increase of $\bar{W}$. Moreover, secant function is an even function, so symmetric behaviour of $\bar{W}$ with respect to vertical axis is obtained. Figure 15 shows the variation in $\bar{W}$ as a function of dimensionless curvature parameter $\bar{\alpha }$ for mirror image of the secant squeeze film bearing. It is observed that $\bar{W}$ increases as $\bar{\alpha }$ moves from 0.2 to 0.6. That means concave shape with more curvature at the centre has more impact on the increase of $\bar{W}$. Again, mirror image of secant is even function, so symmetric behaviour of $\bar{W}$ is obtained.

Patel & Deheri [10] studied squeeze film bearing system formed by upper exponential disc using the Shliomis model with transverse magnetic field. It was shown that $\bar{W}$ increases with the increasing values of $\bar{\beta }$. Also, maximum $\bar{W}$ is obtained about 1.6 when $\bar{\beta }=\text{1.9}$. In the present study, the similar behaviour of $\bar{W}$ is obtained with the advantage of having maximum $\bar{W}$ as 2.24 (refer to tables 2, 3 and 5) at smaller value of $\bar{\beta }=0\text{.6.}$ Shah & Bhat [6] also observed the similar type of increasing behaviour of $\bar{W}$ with the increasing values of $\bar{\beta }$.

Figure 16 shows the comparative study of variation in $\bar{W}$ as a function of dimensionless porous thickness parameter *ψ* for all bearing designs. It is observed that $\bar{W}$ increases in all cases when ψ→0; that is, $\bar{W}$ increases when width of the porous matrix decreases. This may be because of following reason.

According to [16], when porous matrix is attached with any one of the discs, then there is a possibility that the pressure in the porous medium provides a path for the fluid to come out easily to the environment (leakage problem). Of course this varies with permeability of the porous matrix. Thus, the presence of the porous material decreases the resistance to flow in *r*-direction and as a consequence the load-carrying capacity is reduced. The same behaviour also agrees with the theoretical conclusion of the Prakash & Tiwari [18], and experimental results of Wu [19]. The similar type of behaviour is also observed in [10], where they have considered the Shliomis model based on [5] using transverse magnetic field.

Figure 17 shows the comparative study of variation in $\bar{W}$ as a function of dimensionless squeeze velocity parameter *V*_sq_ for all bearing designs. It is observed that squeeze velocity parameter has no effect on $\bar{W}$.

Both figures 16 and 17 show that the secant and parallel squeeze film bearings show almost same behaviour. Moreover, it is observed that $\bar{W}$ is maximum for exponential squeeze film bearing, and minimum for secant and parallel squeeze film bearings with ${\bar{W}}_{e}>{\bar{W}}_{is}>{\bar{W}}_{s}\approx {\bar{W}}_{p}.$ Here, for both figures 16 and 17, $\bar{\beta }=\bar{\alpha }=0.6$ and $\bar{\gamma }=0.2$ are fixed.

## 5. Conclusion

On the basis of ferrohydrodynamic theory by Shliomis and equation of continuity for film as well as porous region, modified Reynolds equation for lubrication of circular squeeze film bearings is derived by considering the effects of oblique radially VMF, slip velocity at the film–porous interface and rotations of both the discs. The squeeze film bearings are made up of circular porous upper disc of different shapes (exponential, secant, mirror image of secant and parallel) and circular impermeable flat lower disc. The validity of Darcy's Law is assumed in the porous region. The FF flow by the Shliomis model is important because it includes the effects of rotations of the carrier liquid as well as magnetic particles. Moreover, the VMF is used because of its advantage of generating maximum field at the required active contact area of the bearing design system. Also, the effect of porosity is included because of its advantageous property of self-lubrication. Using Reynolds equation, pressure equation is derived and expression for dimensionless load-carrying capacity is obtained. Using this expression, results for different bearing design systems are computed and compared for variation of different parameters like rotation, curvature of the upper discs, thickness of the porous matrix and squeeze velocity. The pressure equation derived in the present case is more general in nature and different from all previous studies. Moreover, the present analysis considers the effect of sample magnetic field and it can be extended to other forms of fields similarly. Further, the mirror image of secant design shape is introduced for the first time because such type of shape exists in industry while manufacturing the disc.

The following conclusions can be drawn from the results and discussion:

1. $\bar{W}$ is maximum when $\Omega_{f}=-1$; that is, either $\Omega_{u}$ is rotated in counterclockwise direction and $\Omega_{l}$ in clockwise direction or $\Omega_{u}$ is rotated in clockwise direction and $\Omega_{l}$ in counterclockwise direction with the same speed. But getting $\Omega_{f}=-1$ for faster rotation results moderate reduction in $\bar{W}$.
2. Maximum $\bar{W}$ is obtained in the case of exponential squeeze film bearing while minimum in the case of secant shape with ${\bar{W}}_{e}>{\bar{W}}_{is}>{\bar{W}}_{p}>{\bar{W}}_{s}$.
3. Concave (with respect to lower flat disc) shape of the upper disc with more curvature at the centre has more impact on the increase of $\bar{W}$ when compared with convex shape.
4. Convex (with respect to lower flat disc) shape with less curvature at the centre in upward direction has more impact on the increase of $\bar{W}$.
5. $\bar{W}$ increases even if rotation of the lower disc is zero and irrespective of the decrease of rotation of the upper disc.
6. $\bar{W}$ increases when dimensionless porous thickness parameter (*ψ*) approaches to 0.
7. $\bar{W}$ almost remains constant when squeeze velocity parameter increases.

## Supplementary Material

Data Availability.doc

## Data accessibility

All data discussed are generated results and uploaded as the electronic supplementary material.
