# Supplementary material for: Ferrofluid lubrication of circular squeeze film bearings controlled by variable magnetic field with rotations of the discs, porosity and slip velocity
Source: R Soc Open Sci. 2017 Dec 13;4(12):170254. doi: 10.1098/rsos.170254 (PMC5749983; doi:10.1098/rsos.170254)
Supplement: Data Availability.doc [file rsos170254supp1.doc]

Data of Figure 3

|  | *Ω_f_* | -2 | -1.67 | -1.33 | 1.33 | 1.67 | 2 |
| --- | --- | --- | --- | --- | --- | --- | --- |
|  | w | 2.233135 | 2.236347 | 2.238633 | 2.223556 | 2.217501 | 2.210519 |

Data of Figure 4

| *Ω_f_* | -2 | -1.67 | -1.33 | 1.33 | 1.67 | 2 |
| --- | --- | --- | --- | --- | --- | --- |
| w | 0.527111 | 0.53065 | 0.533172 | 0.516725 | 0.510091 | 0.502439 |

Data of Figure 5

| *Ω_f_* | -2 | -1.67 | -1.33 | 1.33 | 1.67 | 2 |
| --- | --- | --- | --- | --- | --- | --- |
| w | 0.954479 | 0.95793 | 0.960389 | 0.944291 | 0.937809 | 0.930333 |

Data of Figure 6

| *Ω_f_* | -2 | -1.67 | -1.33 | 1.33 | 1.67 | 2 |
| --- | --- | --- | --- | --- | --- | --- |
| w | 0.685525 | 0.689031 | 0.691528 | 0.675212 | 0.668635 | 0.66105 |

Data of Figure 7

| *Ω_f_* | -1 | -0.75 | -0.6 | -0.5 | 0.5 | 0.6 | 0.75 | 1 |
| --- | --- | --- | --- | --- | --- | --- | --- | --- |
| w | 2.239992 | 2.237145 | 2.232947 | 2.227397 | 2.204781 | 2.2141 | 2.222068 | 2.228684 |

Data of Figure 8

| *Ω_f_* | -1 | -0.75 | -0.6 | -0.5 | 0.5 | 0.6 | 0.75 | 1 |
| --- | --- | --- | --- | --- | --- | --- | --- | --- |
| w | 0.534677 | 0.532719 | 0.529613 | 0.525361 | 0.50069 | 0.509054 | 0.516271 | 0.522341 |

Data of Figure 9

| *Ω_f_* | -1 | -0.75 | -0.6 | -0.5 | 0.5 | 0.6 | 0.75 | 1 |
| --- | --- | --- | --- | --- | --- | --- | --- | --- |
| w | 0.961853 | 0.959665 | 0.956277 | 0.951689 | 0.927543 | 0.936156 | 0.943568 | 0.94978 |

Data of Figure 10

| *Ω_f_* | -1 | -0.75 | -0.6 | -0.5 | 0.5 | 0.6 | 0.75 | 1 |
| --- | --- | --- | --- | --- | --- | --- | --- | --- |
| w | 0.693017 | 0.690972 | 0.68776 | 0.683381 | 0.658907 | 0.667365 | 0.674656 | 0.68078 |

Data of Figure 13

| |  | | --- | | -0.6 | -0.4 | -0.2 | 0 | 0.2 | 0.4 | 0.6 |
| --- | --- | --- | --- | --- | --- | --- | --- | --- |
| w | 0.2347 | 0.330907 | 0.475156 | 0.693017 | 1.022379 | 1.516125 | 2.239992 |

Data of Figure 14

| |  | | --- | | -0.6 | -0.4 | -0.2 | 0 | 0.2 | 0.4 | 0.6 |
| --- | --- | --- | --- | --- | --- | --- | --- | --- |
| w | 0.534677 | 0.617499 | 0.673324 | 0.693017 | 0.673324 | 0.617499 | 0.534677 |

Data of Figure 15

| |  | | --- | | -0.6 | -0.4 | -0.2 | 0 | 0.2 | 0.4 | 0.6 |
| --- | --- | --- | --- | --- | --- | --- | --- | --- |
| w | 0.961853 | 0.786582 | 0.713743 | 0.693017 | 0.713743 | 0.786582 | 0.961853 |

Data of Figure 16

| ψ | 0 | 0.0002856 | 0.002856 | 0.02856 |
| --- | --- | --- | --- | --- |
| w_e_ | 2.534811 | 2.5015651 | 2.239992 | 1.121757 |
| w_s_ | 0.695333 | 0.6930686 | 0.673324 | 0.523196 |
| w_os_ | 1.009024 | 1.004094 | 0.961853 | 0.678315 |
| w_p_ | 0.716328 | 0.7139285 | 0.693017 | 0.535132 |

Data of Figure 17

| V | 0.212121 | 0.636363 | 1.060606 | 1.484848 |
| --- | --- | --- | --- | --- |
| w_e_ | 2.239992 | 2.243058 | 2.243671 | 2.243934 |
| w_s_ | 0.673324 | 0.675762 | 0.67625 | 0.676459 |
| w_os_ | 0.961853 | 0.964409 | 0.964921 | 0.96514 |
| w_p_ | 0.693017 | 0.695464 | 0.695953 | 0.696163 |
